# Editorial: *In silico* Modeling of Brain Receptors for Antidepressants, Psychostimulants, and Other CNS-Active Drugs

**DOI:** 10.3389/fphar.2015.00302

**Published:** 2016-01-08

**Authors:** Christopher K. Surratt, Jeffry D. Madura

**Affiliations:** ^1^Mylan School of Pharmacy, Graduate School of Pharmaceutical Sciences, Duquesne UniversityPittsburgh, PA, USA; ^2^Department of Chemistry and Biochemistry, Bayer School of Natural and Environmental Sciences, Center for Computational Sciences, Duquesne UniversityPittsburgh, PA, USA

**Keywords:** *in silico* modeling, computational neuroscience, transporter protein, ion channel gating, molecular dynamics simulations, conformation

The physiological binding sites for antidepressants, antipsychotics, psychostimulants, opiates, and anticonvulsants are embedded in the membranes of neurons and their vesicles. Such integral membrane proteins (IMPs) have been notoriously difficult to crystallize *in situ* (Bolla et al., 2012), and extracting the proteins from the membrane before crystallization may render a nonfunctional protein. For decades, the dearth of precise structural information on IMP receptors and channels in the CNS hindered understanding of their 3D structures and mechanisms of action, as well as development of new medications. Computational tools for *in silico* (“virtual”) modeling of drug receptors have been available since the early 1980s, yet their output could only be as accurate as the known protein structure. More recently, breakthroughs related to IMP structure and function have yielded high-resolution x-ray data for G protein-coupled receptors, voltage- and ligand-gated ion channels, and neurotransmitter transporter proteins. These structures are finally providing credible templates on which to build IMP computational models.

The seven articles in this special issue involve IMPs that control “gating” of substrates and/or ions across the lipid bilayer. Passage of substrates/ions through a hydrophilic pore created by IMP transmembrane helices is dependent on whether the channel is in an “open” or “closed” conformation. These articles reflect the power of today's computational methods to characterize discrete transporter or channel conformations and the mechanisms by which they bind ligands or interconvert between conformations. Characterization of discrete conformations with virtual models is essential to assess substrate/ion translocation, the ligand-receptor interaction, and to predict drug candidates.

Six articles focus on the “secondary active transporter” class of IMPs. Primary active transporters require an energy source such as ATP hydrolysis to drive transmembrane passage of substrates or ions against their concentration gradients (“uphill”). Secondary active transporter proteins, in contrast, move the substrate uphill by simultaneously transporting one or more ion cofactors “downhill,” providing the energy for transport. In the course of the substrate translocation cycle, a transporter conformation with the substrate/ion permeation pore open to the extracellular space (outward-facing; OF) interconverts with a conformation open to the cytoplasm (inward-facing; IF). There may be intermediate conformations as well, depending on the transporter protein.

Five of the six secondary active transporter articles deal with plasma membrane transporters for the monoamine neurotransmitters dopamine, norepinephrine and serotonin, best known as the targets of antidepressant drugs including citalopram (Celexa™) and duloxetine (Cymbalta™). Our understanding of human monoamine transporter (hMAT) structure and function surged forward with crystal structures for the distantly homologous bacterial leucine transporter (LeuT; Yamashita et al., 2005) and the *Drosophila* dopamine transporter (dDAT; Penmatsa et al., 2013) from Eric Gouaux and colleagues. Models presented in all five issue articles employed Gouaux structures as templates. Molecular dynamics (MD) simulations of MAT recognition of substrates, inhibitors and ion cofactors that drive transport, as well as the translocation mechanism itself, are reviewed (Grouleff et al.). MD visualizes each atomic shift of the protein as the external gating residues close behind the entering substrate/ions, triggering opening of the internal gate and movement of substrate and cofactors into the cell. A second review article compares binding of structurally dissimilar substrates and inhibitors in the primary substrate (S1) pockets of dDAT, the three hMATs, and LeuBAT, the latter a LeuT mutant protein in which the S1 pocket residues of LeuT have been replaced with their human serotonin transporter (hSERT) counterparts (Koldsø et al.). The ligand binding orientations in S1 predicted from the models are in close agreement with previously reported *in vitro* binding data.

A dDAT-based homology model of the OF conformation of hDAT was used to predict the activity of a drug never before tested as a DAT ligand (Cheng et al.). Using MD to follow the movement of the anticholinergic drug orphenadrine within the DAT model, the drug behaved much like the competitive DAT inhibitor cocaine, and unlike the DAT substrates dopamine and amphetamine. The MD prediction for orphenadrine was confirmed by *in vitro* pharmacologic and cell biologic assays. In reviewing MAT model-guided drug design (Mortensen and Kortagere), the authors mention their own studies on identifying an allosteric hSERT binding pocket and using the pocket to screen a small molecule virtual library. Hit compounds retrieved by the model were found to modulate SERT interactions with serotonin, antidepressants, and psychostimulants. The emerging field of fragment-based drug design (FBDD) is also reviewed in the context of an hSERT model (as well as a dopamine D3 receptor model) in this issue (Wasko et al.). FBDD here entails the use of “virtual medicinal chemistry” algorithms to build novel ligands from within the receptor binding site. The sixth secondary active transporter article (Vergara-Jaque et al.) differs from the previous five in that the transporter class, represented by the glutamate transporter Glt_Ph_, requires the substrate binding pocket itself to move as a piston, or elevator, while interconverting between the OF and IF conformations. Using repeat-swap homology modeling, the authors successfully generate an OF model of the concentrative nucleoside transporter VcCNT using as template the opposite (IF) conformation of the nonhomologous Glt_Ph_ protein that shares its asymmetrical structure. The final article of the issue explores the hydrophobic effect as a gating mechanism for the K^+^ channel KcsA (Yonkunas and Kurnikova). Virtual KcsA mutations were employed to model the transition between the closed and open channel states.

Virtual screening with IMP models allows for discovery of novel or repurposed drugs unlikely to be found using a conventional SAR approach, providing “needle in a haystack” lead compounds that would take considerably more time and money to find with *in vitro* pharmacologic high-throughput screens. Computational models allow academic laboratories and similar small-budget enterprises to participate in drug discovery. Atomistic and coarse-grained MD simulations allow investigation of IMP conformational changes during receptor activation or substrate/ion movement through a membrane channel. The articles within reflect the cutting edge of *in silico* approaches for understanding brain receptor mechanisms of action, orientation of ligands within the receptor, and virtual discovery of novel lead compound therapeutics.

## Author contributions

CS and JM contributed equally in the writing of this editorial.

## Funding

This work was supported by NIH grant DA027806.

### Conflict of interest statement

The authors declare that the research was conducted in the absence of any commercial or financial relationships that could be construed as a potential conflict of interest.
